# The budding yeast GSK-3 homologue Mck1 is an essential component of the spindle position checkpoint

**DOI:** 10.1098/rsob.220203

**Published:** 2022-11-02

**Authors:** Siddhi Rathi, Irem Polat, Gislene Pereira

**Affiliations:** ^1^ Centre for Organismal Studies (COS), University of Heidelberg, Heidelberg, Germany; ^2^ Heidelberg Biosciences International Graduate School (HBIGS) and Faculty of Biosciences, University of Heidelberg, Heidelberg, Germany; ^3^ Centre for Molecular Biology (ZMBH), University of Heidelberg, Heidelberg, Germany; ^4^ German Academic Exchange Service (DAAD), Bonn, Germany; ^5^ German Cancer Research Centre (DKFZ), DKFZ-ZMBH Alliance, Heidelberg, Germany

**Keywords:** cell division, checkpoint control, mitotic exit, budding yeast, MCK1, Cdc6

## Abstract

The spindle position checkpoint (SPOC) is a mitotic surveillance mechanism in *Saccharomyces cerevisiae* that prevents cells from completing mitosis in response to spindle misalignment, thereby contributing to genomic integrity. The kinase Kin4, one of the most downstream SPOC components, is essential to stop the mitotic exit network (MEN), a signalling pathway that promotes the exit from mitosis and cell division. Previous work, however, suggested that a Kin4-independent pathway contributes to SPOC, yet the underlying mechanisms remain elusive. Here, we established the glycogen-synthase-kinase-3 (GSK-3) homologue Mck1, as a novel component that works independently of Kin4 to engage SPOC. Our data indicate that both Kin4 and Mck1 work in parallel to counteract MEN activation by the Cdc14 early anaphase release (FEAR) network. We show that Mck1's function in SPOC is mediated by the pre-replication complex protein and mitotic cyclin-dependent kinase (M-Cdk) inhibitor, Cdc6, which is degraded in a Mck1-dependent manner prior to mitosis. Moderate overproduction of Cdc6 phenocopies *MCK1* deletion and causes SPOC deficiency via its N-terminal, M-Cdk inhibitory domain. Our data uncover an unprecedented role of GSK-3 kinases in coordinating spindle orientation with cell cycle progression.

## Graphical abstract

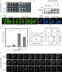

## 1. Introduction

Every cell division in *Saccharomyces cerevisiae* is asymmetric and gives rise to a new daughter and an old mother cell. Yeast cells polarize at early stages of the cell cycle to orient the spindle formed in the mother compartment along the mother–daughter polarity axis. The mitotic spindle position checkpoint (SPOC) ensures faithful segregation of chromosomes by sensing mitotic spindle orientation [1].

To exit mitosis and prepare for the next cell cycle, cells inactivate the mitotic cyclin-dependent kinase (M-Cdk) complex and dephosphorylate its substrates [2,3]. This inactivation is driven by a conserved proline-directed protein phosphatase, Cdc14, which is retained in the nucleolus through most of the cell cycle [4,5]. During anaphase, Cdc14 is released into the nucleus and cytoplasm primarily in two waves. First, a partial release of Cdc14 from the nucleolus into the nucleus is achieved by the Cdc14 early anaphase release (FEAR) network at the metaphase–anaphase transition [6–8]. This is followed by activation of the mitotic exit network (MEN) that promotes the full release of Cdc14 into the cytoplasm at the end of anaphase [5,9–11]. The pool of Cdc14 released by the FEAR network is required for accurate chromosome partitioning and spindle formation, however, this Cdc14 pool is insufficient to drive mitotic exit [12–14]. For this, the full release of Cdc14 by the MEN is indispensable [4,15–18].

The MEN consists of the Ras-like small GTPase Tem1 that when bound to the nucleotide GTP activates a downstream kinase cascade composed of Hippo-like kinase Cdc15 and the LATS/NDR kinase Dbf2–Mob1 complex [6,19–21]. Upon Tem1 activation, Cdc15 is recruited to the spindle pole body (SPB, the functional equivalent of mammalian centrosome) where it activates Dbf2–Mob1 [20–23]. The polo-like kinase Cdc5 targets active Dbf2–Mob1 to the nucleolus where it phosphorylates the Cdc14-anchor, Cfi1/Net1, and Cdc14 [5,24]. This leads to the release of active Cdc14 into the nucleoplasm and cytoplasm [5,25]. Ultimately, the timing of mitotic exit is governed by the full release of Cdc14 phosphatase that counteracts M-Cdk activity via Clb2 (mitotic cyclin) degradation and stabilization of the Cdk inhibitor Sic1. The MEN and Cdc14 also promote cytokinesis by dephosphorylating key Cdk targets [26,27]. In addition to Sic1, Cdh1/Hct1 and Cdc6 cooperate to downregulate M-Cdk activity during late anaphase [28]. Cdh1/Hct1 is a regulatory subunit of E3 ubiquitin ligase complex that recognizes Clb2 and aids M-Cdk inactivation [29,30]. Whereas, Cdc6 is a pre-replicative complex (pre-RC) component that is shown to inhibit M-Cdk activity in a manner that is unrelated to its role in DNA replication [28,31,32].

SPOC prevents mitotic exit until its anaphase spindle is correctly oriented along the mother–daughter polarity axis by inhibiting the MEN on the level of Tem1 [1,33,34]. Under unperturbed conditions, the bipartite Bfa1–Bub2 GTPase-activating protein (GAP) complex inhibits Tem1 activation until Bfa1 at SPBs becomes phosphorylated by Cdc5 in cells with properly aligned spindles [35]. Upon spindle misalignment, the SPOC kinase Kin4 phosphorylates Bfa1 at SPBs thereby recruiting the 14-3-3 family protein Bmh1 [33–36]. This leads to the disassociation of Bfa1–Bub2 complex from the SPBs and sustained Tem1 inhibition [17,36–42].

Recent studies provided evidence of a Kin4-independent pathway that holds the SPOC arrest parallel to the established Kin4-dependent regulation [43–45]. In the absence of FEAR, Bfa1 phosphorylation by Kin4 is dispensable to hold mitotic arrest upon spindle misalignment suggesting that mechanisms in addition to Kin4 link spindle orientation to MEN regulation [42,46]. Recently, it was described that the dephosphorylation of Bfa1 by the Glc7-Bud14 kinase complex serves as Kin4-independent mechanism to keep Bfa1–Bub2 active in cells with misaligned anaphase spindles by counteracting Cdc5 phosphorylation [45]. However, whether spindle orientation defects can feed directly into maintaining M-Cdk activity in addition to the regulation of Bfa1–Bub2 or MEN signalling remains an open question.

In this study, we established Mck1, one of the four GSK-3 protein kinase homologues in yeast, as a novel SPOC component and mitotic exit inhibitor. We show that Mck1 is essential to halt mitotic exit in cells with misaligned spindles in parallel to the Kin4 SPOC pathway. Additionally, Mck1 functions in mitosis by targeting the pre-RC protein Cdc6 for degradation. Lack of Mck1 activity bypasses Kin4 hyperactivity and causes a SPOC defect in cells with anaphase spindle misalignment. Our study provides insight into the function of Mck1 in spindle alignment and mitotic exit.

## 2. Results

### 2.1. Deletion of *MCK1* rescues the lethality of *KIN4* overexpression and causes SPOC deficiency

**Figure 1.**
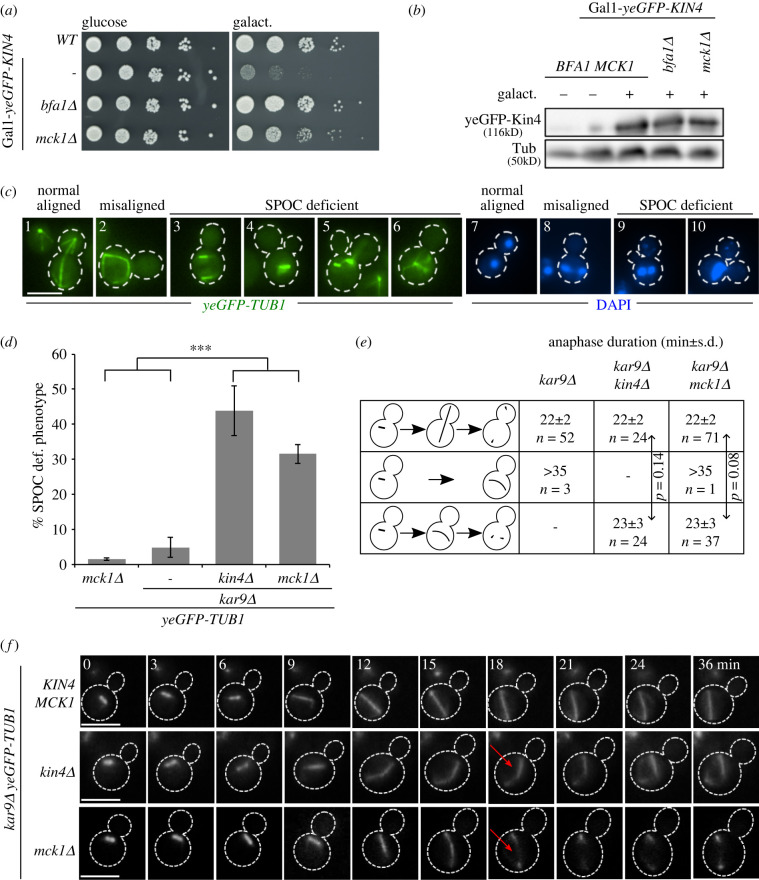
Mck1 is a novel SPOC component. (*a*) Serial dilutions of the indicated strains were spotted on the Gal1-*KIN4* repressing (glucose) and Gal1-*KIN4* inducing agar plates containing galactose (galact.) and incubated at 30°C for 3 days. (*b*) Immunoblot shows expression levels of yeGFP-Kin4 in the presence and absence of galactose as indicated. Tubulin (Tub) served as a loading control. (*c,d*) Representative images of SPOC deficient cells (*c*) and SPOC analysis of strains are indicated in (*d*). Cells were fixed and analysed for yeGFP-tubulin and DNA content by DAPI staining. Dashed lines mark the cell boundary. SPOC deficient phenotypes were scored in (*d*) based on tubulin staining (*n* = 100 cells per strain and experiment). The graph depicts average ± s.d. of SPOC deficient phenotypes from three independent experiments. Asterisk indicates a significant difference based on the two-tailed Student's *t*-test, *p* < 0.05 (*), *p* < 0.001 (**) and *p* < 0.0001 (***). (*e,f*) SPOC analysis by live cell imaging. Anaphase duration (*e*) and still images (*f*) are shown for *kar9Δ* (*n* = 55), *kar9Δ kin4Δ* (*n* = 48) and *kar9Δ mck1Δ* (*n* = 109) cells. The quantifications in (*e*) show anaphase duration (mean ± s.d.) for cells with normal or misaligned spindles as depicted. Still images in (*f*) show cells in which the spindle elongated in the mother cell body; *t* = 0 was defined as the time prior to the beginning of spindle elongation. Cell boundaries are marked by dashed lines and red arrow indicates spindle disassembly. Scale bars, 3 µm.

Overexpression of *KIN4* leads to arrest of cells in late anaphase due to constant inactivation of the MEN GTPase Tem1 by Bfa1–Bub2 GAP complexes [33]. In a genome-wide screen carried out to identify novel SPOC components or mitotic exit inhibitors, *MCK1* was identified as a gene whose deletion bypasses the toxic effects of *KIN4* overexpression [42]. To validate the screen, we analysed the growth of *MCK1* and *mck1Δ* cells that carried an extra copy of *KIN4* under control of the galactose inducible promoter (Gal1-*KIN4*). Under galactose-inducing conditions, *mck1Δ* cells survive the toxicity of *KIN4* overexpression similar to cells bearing *BFA1* deletion (figure 1*a*). The levels of Kin4 upon induction were comparable in both *MCK1* and *mck1Δ* strains (figure 1*b*), excluding an effect of Mck1 upon Kin4 protein stability. Together, we concluded that Mck1 either regulates Kin4 activity, acts downstream or parallel to Kin4 to inhibit mitotic exit.

To examine if Mck1 is required for SPOC function, we made use of cells lacking the adenomatous polyposis coli-related spindle-positioning factor Kar9 that is required for spindle orientation [47]. In the case of SPOC dysfunction, cells with misaligned spindles accumulate multi-budded and multinucleated cells as assessed by nuclear and tubulin staining with DAPI and yeast-enhanced (ye)*GFP*, respectively (figure 1*c*) [40,48]. SPOC deficiency was observed for the majority of *kar9Δ mck1Δ* but not *kar9Δ* or *mck1Δ* cells (figure 1*c* and *d*).

To verify that cells lacking Mck1 cannot engage the SPOC, we next determined the duration of anaphase (defined as the time from the start of spindle elongation until spindle disassembly) in *kar9Δ mck1Δ* ye*GFP-TUB1* cells by live-cell imaging. As controls, we used *kar9Δ* ye*GFP-TUB1* (SPOC proficient) and *kar9Δ kin4Δ* ye*GFP-TUB1* (SPOC deficient) cells. In all cell types, when the spindle was normally aligned, the anaphase duration was approximately 22 min (figure 1*e,f*). In *kar9Δ* cells, the misaligned spindle stayed intact during the time of inspection and did not disassemble. Contrary to this, we observed that in most *kar9Δ mck1Δ* cells the time taken by the misaligned anaphase spindle to disassemble in the mother cell compartment did not significantly vary in comparison to cells with a correctly aligned spindle (figure 1*e,f*–arrowheads). The behaviour of *kar9Δ mck1Δ* cells was similar to *kar9Δ kin4Δ* cells (figure 1*e,f*), indicating SPOC deficiency. Together, Mck1 is a SPOC component essential to stall mitotic exit in cells with a misaligned anaphase spindle. Previously, Mck1 was predicted to regulate spindle positioning via modulation of astral microtubule dynamics [49]. However, we did not observe any spindle orientation defects in *mck1Δ* cells (electronic supplementary material, figure S1A). This implies that Mck1 does not affect spindle positioning in our strain background and growth conditions.

**Figure 2.**
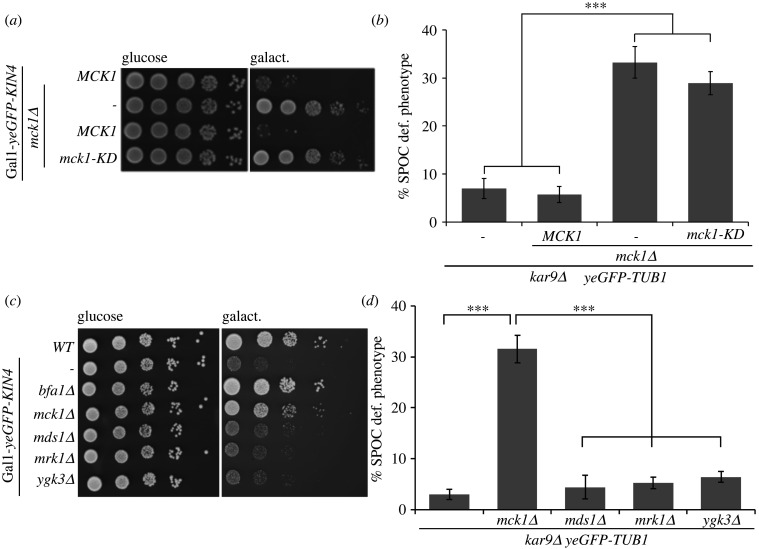
Mck1 kinase activity is vital for its SPOC function. (*a*) Serial dilutions of Gal1-*yeGFP-KIN4 MCK1* and Gal1-*yeGFP-KIN4 mck1Δ* cells carrying an empty plasmid (pRS315), pRS315-*MCK1* or pRS315-*mck1*-kinase dead (KD) as indicated. Growth is shown on Gal1-*KIN4* repressing (glucose) or inducing (galact.) selection plates after 3 days at 30°C. (*b*) Percentage of SPOC-deficient phenotypes for the indicated strains carrying an integrated copy of *MCK1* and *mck1-KD*. Average ± s.d. of three independent experiments (*n* = 100 anaphase cells per strain and experiment) is shown. (*c*) Serial dilutions of wild-type and Gal1-*yeGFP-KIN4* strains carrying the indicated gene deletions on glucose (Gal1 repressing) and galactose (Gal1 inducing) plates. Growth is shown after 3 days at 30°C. (*d*). SPOC-deficient phenotypes of indicated strains. Graph shows average ± s.d. of three independent experiments (*n* = 100 cells per strain and experiment). Asterisks in (*b*) and (*d*) indicate a significant difference based on the two-tailed Student's *t*-test, *p* < 0.05 (*), *p* < 0.001 (**) and *p* < 0.0001 (***).

Next, we questioned if the kinase activity of Mck1 is essential for the SPOC function. We used a kinase-dead version of *MCK1*, *mck1-KD*, in which aspartic acid at amino acid position 164 was mutated to alanine [50]*.* In the *KIN4* overexpression background, *mck1-KD* cells behaved like *mck1Δ* and were able to grow under Kin4 overproducing conditions (figure 2*a*). Furthermore, *kar9Δ mck1-KD* cells were SPOC deficient similar to *kar9Δ mck1Δ* cells (figure 2*b*). We concluded that Mck1 kinase activity is essential to regulate SPOC. The SPOC function of Mck1 appears to be unique to it as the deletion of the three other GSK3 homologues, Mds1 (Rim11), Mrk1 and Ygk3, did not rescue Kin4 overproducing lethality or caused SPOC deficiency upon spindle misorientation (figure 2*c*,*d*).

### 2.2. Mck1 does not influence Kin4 activity

We reasoned that Mck1 might regulate Kin4 to control SPOC. To test this possibility, we analysed Kin4 localization and activity *in vivo* in the presence and absence of *MCK1*. The role of Kin4 in SPOC depends upon its localization to the mother cell cortex and SPBs [33,34,41]. We, therefore, looked at the localization of Kin4 upon spindle misalignment in anaphase cells lacking Mck1. Our data show that Kin4 associates with both the SPBs in *kar9Δ mck1Δ* cells with mispositioned spindles (electronic supplementary material, figure S1B-c,d), similar to what has been reported previously for *kar9Δ* cells [34] (electronic supplementary material, figure S1B-a,b). To further analyse Kin4 localization, wild-type and *mck1Δ* cells carrying *KIN4-mNeonGreen* and the SPB marker *SPC42-mCherry* were treated with the microtubule depolymerizing drug nocodazole, which arrests cells in metaphase in a Bfa1–Bub2 dependent manner [34] (electronic supplementary material, figure S1C). In both, wild-type and *mck1Δ* cells, Kin4-mNeonGreen associated with the mother cell cortex and SPBs (electronic supplementary material, figure S1D) confirming that Mck1 does not control Kin4 localization.

To evaluate Kin4 activity, we assessed the phosphorylation profile of Bfa1 by monitoring Bfa1 mobility shift on SDS-PAGE gels. It is known that Kin4 phosphorylates Bfa1 to prevent Bfa1 from the inhibitory phosphorylation by polo-like kinase Cdc5 [33,34]. In nocodazole-treated cells, the slowly migrating forms of Bfa1, which are phosphorylated by Cdc5, become apparent only upon deletion of *KIN4* [34,36,41,51,52] (electronic supplementary material, figure S1E, *kin4Δ* cells, asterisk). These slow-migrating forms of Bfa1 were absent in *mck1Δ* cells, resembling *KIN4 MCK1* cells (electronic supplementary material, figure S1E). Importantly, the deletion of *KIN4* in *mck1Δ* background promoted hyperphosphorylation of Bfa1 (electronic supplementary material, figure S1E) and supported the conclusion that Kin4 and Cdc5 are both active in the absence of Mck1.

Phospho-regulation of Bfa1 by Kin4 and Cdc5 governs the SPB localization of the Bfa1–Bub2 GAP complex [34,35]. Upon SPOC activation, phosphorylation of Bfa1 by Kin4 removes Bfa1–Bub2 from SPBs thereby changing the SPB localization of the complex from asymmetric (stronger signal at one SPB) to symmetric (similar levels at both SPBs) [37,38]. To further confirm Kin4 function, we determined Bfa1 SPB localization in *mck1Δ* cells upon spindle misalignment. As reported earlier, Bfa1 localized symmetrically in the majority of *kar9Δ* cells with misoriented spindles (electronic supplementary material, figure S1F) [37]. By contrast, Bfa1 localized asymmetrically at the SPBs in *kar9Δ kin4Δ* cells (electronic supplementary material, figure S1F) [37,38], upon spindle misalignment. Notably, we see that *kar9Δ mck1Δ* cells show Bfa1 localization behaviour similar to that of the *kar9Δ* cells (electronic supplementary material, figure S1F). Together, these data strongly indicate that Mck1 does not regulate the localization or activity of Kin4 *in vivo*.

### 2.3. Deletion of *MCK1* leads to MEN activation in cells with misaligned spindles

**Figure 3.**
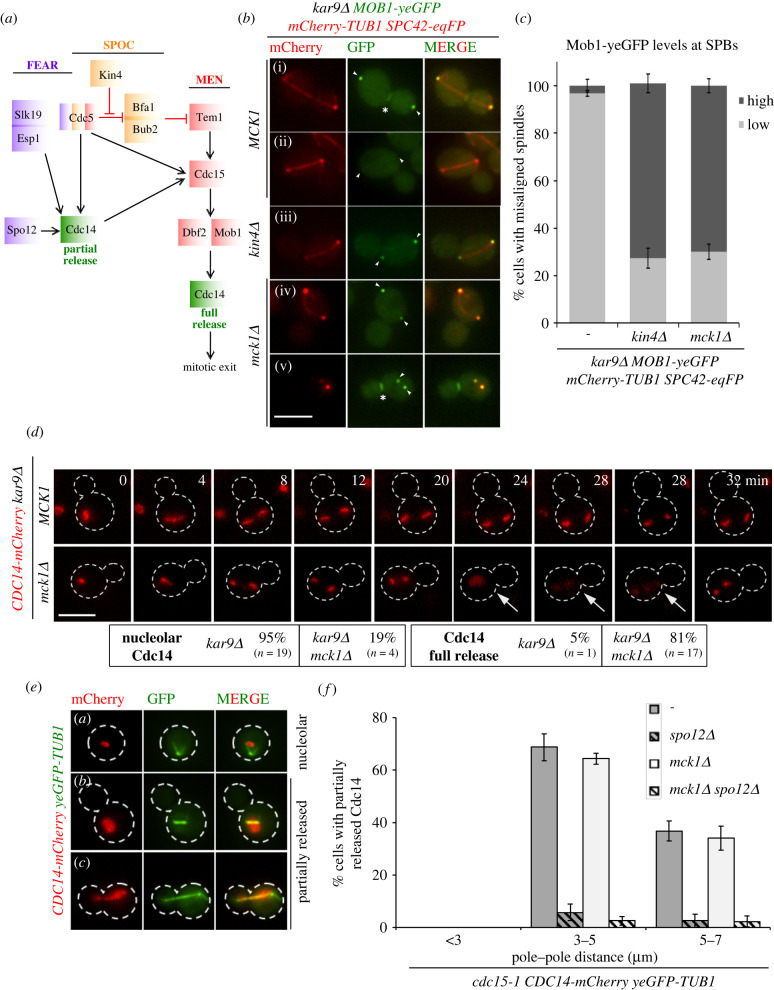
*mck1Δ* cells with misaligned spindles activate MEN. (*a*) The illustration represents a flow chart of the interplay between key components of the FEAR (purple), SPOC (orange) and MEN (red) pathway in order to achieve Cdc14 (green) partial or full release. The red capped lines and black arrows indicate inhibitory and activating events. (*b*) Representative images show Mob1–yeGFP localization at SPBs (labelled with Spc42-eqFP) in cells with properly aligned (*a*) and misaligned (*b–e*) spindles. mCherry-Tub1 served as a spindle marker. Arrowheads and asterisks indicate SPBs and bud neck, respectively. Please note, in panel (e) the two close SPBs, the absence of spindle microtubules and Mob1–yeGFP accumulation at SPBs and bud neck indicate mitotic exit upon spindle misalignment. Scale bar, 3 µm. (*c*) Graph represents the percentage of cells with misaligned spindles harbouring high and low Mob1–yeGFP at SPBs. Average ± s.d. of three independent experiments is shown. *N* = 100 cells with misaligned spindles per strain and per experiment. (*d*) Representative still images from live-cell imaging showing Cdc14-mCherry localization in *kar9Δ MCK1* and *kar9Δ mck1Δ* cells with misaligned spindles. Cell boundaries are marked by dashed lines; the arrows indicate Cdc14 full release. Scale bars, 3 µm. The percentage of cells with nucleolar or fully released Cdc14 upon spindle misalignment is mentioned. (*e,f*) Analysis of FEAR-network dependent release of Cdc14. Cells were arrested at 23°C in the G1 phase with alpha-factor and released at 37°C in the absence of alpha-factor. Samples were taken every 15 min for 150 min. Cdc14 full and partial release was scored based on spindle length (pole–pole distance). Representative images in (*e*) show examples of cells with nucleolar or partially released Cdc14-mCherry. Cell boundaries are marked by dashed lines and yeGFP-Tub1 served as a spindle marker. Graph in (*f*) show the percentage of cells with partially released Cdc14-mCherry for the indicated strains and category. Average ± s.d. from three independent experiments is shown. For each strain, 100 cells per category and experiment were counted. Scale bars, 3 µm.

We next asked whether Mck1 contributes to SPOC by blocking MEN activation at SPBs and Cdc14 nucleolar release. A stereotypical behaviour of MEN components, including Cdc15 and Mob1–Dbf2 kinases, is their recruitment to SPBs upon Tem1 GTPase activation [20–23,53] (figure 3*a*). Mob1–Dbf2 also binds to the bud neck (the site of cytokinesis) in late anaphase after downregulation of M-Cdk activity [22,23] (figure 3*b*(i); asterisk). As expected, Mob1–yeGFP accumulated at SPBs and bud neck in late anaphase *MCK1 kar9Δ* cells with correctly aligned but not misaligned spindles (figure 3*b*ii, figure 3*c*) [42]. The percentage of cells with spindle misorientation and Mob1–yeGFP at SPBs (figure 3*b*iv, arrowheads) or bud neck (figure 3*b*v, asterisk) largely increased in the absence of *MCK1* (figure 3*c*). This behaviour was comparable to *kin4Δ* (figure 3*b*iii, figure 3*c*) and implied that Mck1 blocks MEN activation when the SPOC is engaged. A consequence of MEN activation is the full release of the phosphatase Cdc14 out of the nucleolus in late anaphase [4,18]. We postulated that deletion of *MCK1* would allow Cdc14 full release in spite of spindle elongation in the mother cell. Indeed, in contrast to *kar9Δ*, the majority of *kar9Δ mck1Δ* cells with misoriented spindles released Cdc14 (figure 3*d*). As a consequence of cell cycle progression, Cdc14 was sequestered back to the nucleolus in cells with misaligned spindles lacking Mck1 [54] (figure 3*d*, 32 min). Our data together validate that MEN becomes fully activated in the absence of *MCK1*.

### 2.4. Mck1 becomes dispensable for SPOC in the absence of FEAR

After the metaphase to anaphase transition, Cdc14 is released from the nucleolus into nucleoplasm by the FEAR network [8] (figure 3*a*). As deletion of *MCK1* promoted MEN activation, we reasoned that Mck1 would specifically interfere with MEN but not FEAR-released Cdc14. To test this hypothesis, we analysed Cdc14 release by FEAR in the presence or absence of Mck1. To stop the contribution of MEN for Cdc14 release, we used cells carrying the temperature-sensitive allele, *cdc15-1*. At the restrictive temperature, *cdc15-1* transit through mitosis with an active FEAR but arrest in late anaphase due to MEN blockage [15,17] (figure 3*e*). In *cdc15-1* cells, partially released Cdc14-mCherry became dispersed in the nucleus in large-budded cells as a consequence of FEAR activation (figure 3*e*). In both *MCK1* and *mck1Δ* cells, Cdc14 partial release was observed to similar levels (figure 3*e,f*). This partial release did not occur when *SPO12* was deleted (figure 3*e,f*) confirming FEAR dependency. Thus, the FEAR pathway is active in the absence of Mck1.

It has been established that upon spindle misorientation Kin4 blocks MEN activation by the FEAR network [42,43]. To test whether Mck1 like Kin4 becomes dispensable for SPOC function in FEARless cells, we compared SPOC proficiency in the presence or absence of Spo12. The deletion of *SPO12* completely reverted the SPOC deficiency of *kar9Δ mck1Δ* resembling the behaviour observed for *kar9Δ kin4Δ* cells (electronic supplementary material, figure S2A). We conclude that Mck1 also counteracts FEAR-dependent MEN activation to engage SPOC.

### 2.5. Mck1 is a mitotic exit inhibitor in cells with compromised MEN activity

**Figure 4.**
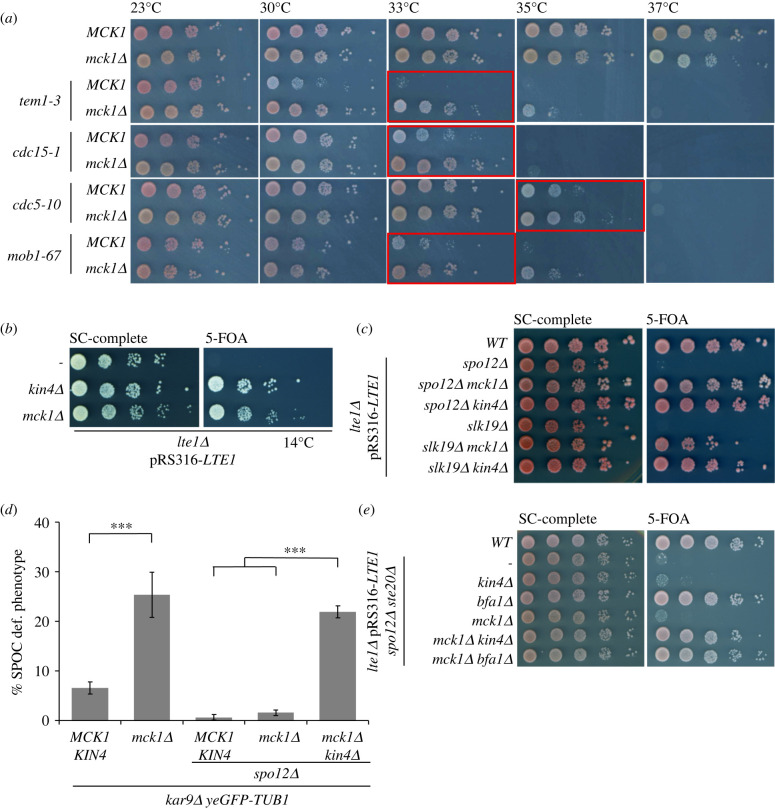
Suppression of mitotic exit defects by *MCK1* deletion. (*a*) Effect of *MCK1* or *KIN4* deletion on the growth phenotype of temperature-sensitive MEN mutants at the indicated temperatures. (*b*) *MCK1* or *KIN4* deletion rescues the cold sensitive growth defect of *lte1Δ* cells. Cells carried chromosomal *LTE1* deletion and a *URA3*-based plasmid expressing *LTE1* (pRS316-*LTE1*). Growth in the presence (SC-complete) or absence (5-FOA) of pRS316-*LTE1* was analysed after 7 days of incubation at 14°C. (*c*) Growth of the indicated strains in the presence (SC-complete) or absence (5-FOA) of pRS316-*LTE1* was analysed after 3 days at 30°C. (*d*) Graph shows the percentage of SPOC-deficient phenotypes for the indicated strains. Average ± s.d. of three independent experiments is shown. *N* = 100 anaphase cells per strain and experiment. Asterisk indicates a significant difference based on the two-tailed Student's *t*-test, *p* < 0.05 (*), *p* < 0.001 (**) and *p* < 0.0001 (***). (*e*) Growth of the indicated strains analysed as in (*c*).

Next, we aimed to investigate whether Mck1 inhibits mitotic exit in cells with properly aligned spindles too. Mck1 was previously shown to delay mitotic entry [50]. In our strain background, the deletion of *MCK1* indeed delayed Clb2 accumulation by 15 min, but only when cells were cultured at 23°C and not 30°C (electronic supplementary material, figure S2B). Under both growth conditions, however, the mitotic duration of *MCK1* and *mck1Δ* cells was comparable (electronic supplementary material, figure S2B). We thus reasoned that if Mck1 plays an inhibitory role in mitosis, this might only become apparent when MEN activity is compromised. To test this hypothesis, we used ways to block mitotic exit other than causing spindle misorientation. Temperature-sensitive mutants of MEN components, including Tem1, Cdc14, Cdc15, Cdc5 and Mob1, delay or completely block mitotic exit in a temperature-dependent manner [18,19] (figure 4*a*). The deletion of *MCK1* was able to rescue the growth of *tem1-3*, *cdc15-1*, *cdc5-10* and *mob1-67* at semi-permissive temperatures (30–35°C). This rescue however required residual MEN activity, as deletion of *MCK1* was not able to promote the growth of mutants at their restrictive temperature (37°C) or rescue *MOB1*, *CDC15* or *TEM1* gene deletions (electronic supplementary material, figure S2C).

Another way to block mitotic exit is via modulation of daughter-cell-specific MEN regulators. In cells with properly aligned spindles, MEN activation requires daughter-cell confined components such as Lte1, a Kin4 inhibitor and putative Tem1 activator [52,55,56]. The deletion of *LTE1* causes growth lethality at cold temperatures (figure 4*b*, 14°C) due to the inability of cells to exit mitosis [56]. This cold lethal growth phenotype can be rescued through deletion of negative regulators of mitotic exit, including *KIN4* (figure 4*b*), *BUB2* or *BFA1* [52,55]. Similarly, deletion of *MCK1* was able to rescue the cold-sensitive growth defect of *lte1Δ* cells (figure 4*b*). As the FEAR network facilitates MEN activation, *LTE1* mutations not only lead to cold sensitivity but also synthetic lethality in the absence of FEAR factors *SPO12 or SLK19* [6]. Similar to *KIN4*, *BFA1* or *BUB2* deletion, cells lacking Mck1 rescued the lethality of *lte1Δ spo12Δ* and *lte1Δ slk19Δ* (figure 4*c*) [6,33]. These data together reinforce the notion that Mck1 acts as a mitotic exit inhibitor.

### 2.6. Mck1 and Kin4 work in parallel to prevent mitotic exit

In cells devoid of FEAR, Kin4 but not Bfa1 becomes dispensable for SPOC, reinforcing the idea that components other than Kin4 prevent mitotic exit. To test whether Mck1 could control mitotic exit in parallel to Kin4, we reassessed SPOC proficiency of *kar9Δ* cells lacking FEAR. As previously shown (electronic supplementary material, figure S2A), deletion of either *MCK1* or *KIN4* did not affect SPOC proficiency of *kar9Δ spo12Δ* cells [42] (figure 4*d*; electronic supplementary material, figure S2A). Strikingly, *mck1Δ kin4Δ* double deletions led to a significant increase in the percentage of SPOC deficient cells (figure 4*d*), implying that Kin4 and Mck1 independently promote SPOC in the absence of FEAR.

To obtain further evidence that Kin4 and Mck1 work together to block MEN, we performed epistasis analysis using a triple deletion mutant background that could be rescued by deletion of *BFA1* but not *KIN4*. A recent study showed that Ste20 promotes mitotic exit parallel to Lte1 and may function upstream of the Bfa1–Bub2 GAP complex [42]. Deletion of *STE20* in combination with *LTE1* and *SPO12* led to lethality that was rescued by deletion of *BFA1* but not *KIN4* [42] (figure 4*e*). Deletion of *MCK1* could not rescue the lethality of *lte1Δ spo12Δ ste20Δ* cells (figure 4*e*). However, when *KIN4* and *MCK1* were deleted in this triple deletion background, cells survived resembling the growth of *lte1Δ spo12Δ ste20Δ bfa1Δ* cells (figure 4*e*). These results together imply that Mck1 and Kin4 block mitotic exit independently of each other but in a redundant manner.

### 2.7. Cdc6 is stabilized in *mck1Δ* cells with normal and misaligned spindles

**Figure 5.**
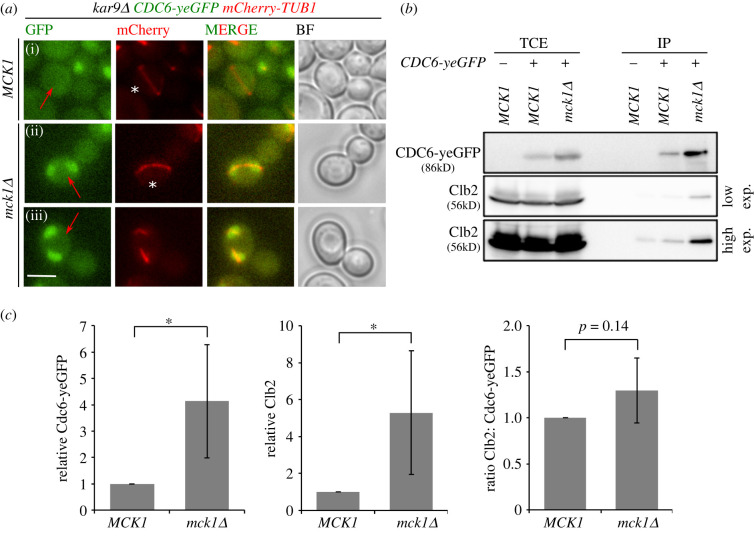
Mck1 inhibits Cdc6 accumulation in mitosis irrespectively of spindle alignment. (*a*) Representative images show Cdc6-yeGFP localization in cells with misaligned spindles (indicated by red arrows) before (i, ii asterisk) or after spindle disassembly (iii). mCherry-Tub1 served as a spindle marker. Scale bar, 3 µm. (*b*) Immunoblot shows the co-immunoprecipitation of Cdc6-yeGFP with Clb2 in *MCK1* and *mck1Δ* cells arrested in mitosis with nocodazole. Low and high exposure time for Clb2 blots is shown. TCE, total cell extract; IP, immunoprecipitated fraction. (*c*) Quantification of (*b*) showing average ± s.d. of four independent experiments. Asterisk indicates a significant difference based on the two-tailed Student's *t*-test, *p* < 0.05 (*), *p* < 0.001 (**) and *p* < 0.0001 (***).

Next, we aimed to elucidate which substrate of Mck1 is involved in SPOC. Cdc6 was one such established substrate of Mck1 [50,57,58] and interesting candidate as it is implicated in mitotic exit regulation apart from its role in DNA replication [28,31,57]. The levels of Cdc6 are tightly regulated to prevent multiple rounds of DNA replication per cell cycle. Phosphorylation of Cdc6 by Mck1 creates a phospho-degron that direct Cdc6 for degradation by the ubiquitin–ligase complex SCF^CDC4^ from G1/S until late mitosis when *CDC6* transcription is upregulated [57–63]. Therefore, cells lacking Mck1 show mitotic accumulation of Cdc6 [57]. We monitored Cdc6 localization in a spatio-temporal manner in our strains. As reported earlier [63], in wild-type cells Cdc6 was degraded after the onset of S-phase and appeared in the nucleus only in late mitotic and G1 cells ([63] electronic supplementary material, figure S3A-a,b). On the contrary, *mck1Δ* cells exhibited localization of Cdc6 throughout the cell cycle [57] (electronic supplementary material, figure S3A-c,d *mck1Δ,* red arrows; S3B). The elevated protein levels of Cdc6 during mitosis was also confirmed by immunoblotting analysis (electronic supplementary material, figure S3C) [57]. Importantly, accumulation of Cdc6 in cells with misaligned spindles was observed in *kar9Δ mck1Δ* but not in *kar9Δ MCK1* (figure 5*a*(i–iii), red arrows), implying that Mck1 governs Cdc6 degradation irrespectively of spindle orientation.

### 2.8. Cdc6–Clb2 complexes accumulate in mitotic cells in the absence of Mck1

*CDC6* is transcribed in late mitosis owing to the presence of an early cell cycle box in its promoter [64–66]. This mitotically expressed Cdc6 associates with M-phase cyclin, Clb2, via its N-terminal ^47^LxF^49^ motif, thereby stabilizing Cdc6 in late mitosis [28,32,67–69]. Binding of Cdc6 to Clb2 and Cdk was shown to inhibit M-Cdk activity *in vivo* and *in vitro* [28,32,70]. We assumed that Cdc6 that accumulates in *mck1Δ* cells associated with more Clb2 molecules in mitotic cells. To test this hypothesis, we immuno-precipitated Cdc6-yeGFP from mitotically arrested cells. We observed a higher proportion of Clb2 in the immuno-precipitates of *mck1Δ* compared to wild-type cells, although the overall Clb2 levels were very similar in both cell types (figure 5*b*,*c*). When comparing the Clb2:Cdc6-yeGFP ratio in the immuno-precipitations, we did not detect any significant difference (figure 5*c*), indicating that the higher amounts of Cdc6 in *mck1Δ* cells proportionally pulls down more Clb2. Altogether we conclude that in *mck1Δ* cells*,* a higher fraction of M-Cdk complexes are inhibited by Cdc6 in the absence of Mck1.

### 2.9. *CDC6* moderate overexpression promotes mitotic exit and SPOC deficiency in an N-terminal domain-dependent manner

**Figure 6.**
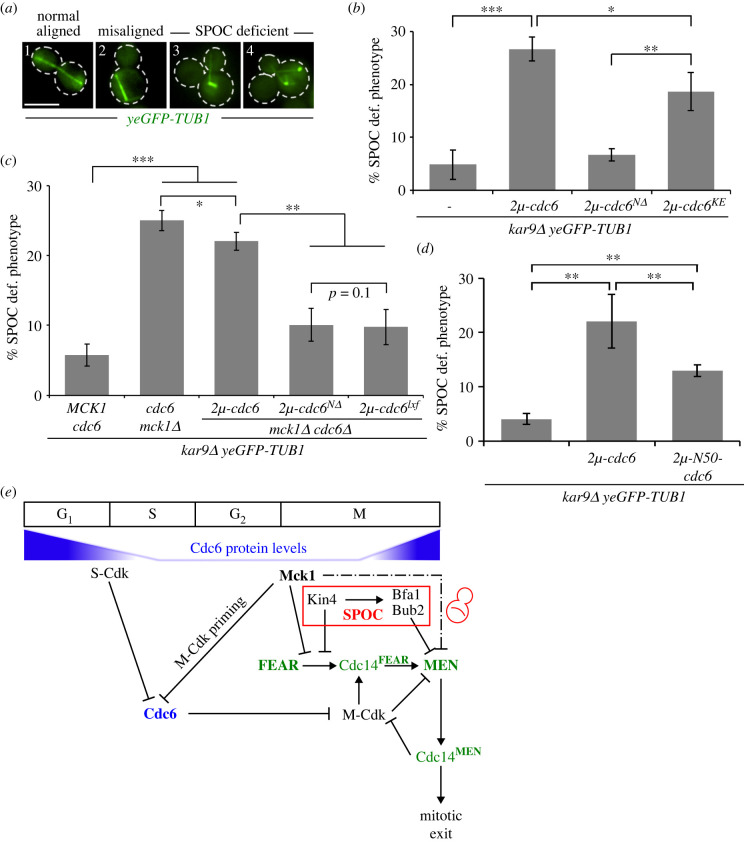
The N-terminal domain of Cdc6 inhibits mitotic exit in cells with misaligned spindle. (*a*) Representative images of cells that were fixed and analysed for yeGFP-tubulin. Scale bar, 3 μm. (*b–d*) Analysis of SPOC in *kar9Δ yeGFP-TUB1* strains carrying high-copy 2µ-plasmids expressing *CDC6* full length, truncated forms or mutants as indicated. Graphs show the average ± s.d. of the percentage of cells with SPOC deficient phenotypes scored from three independent experiments. *N* = 100 anaphase cells per strain and experiment. Asterisk indicates a significant difference based on the two-tailed Student's *t*-test, *p* < 0.05 (*), *p* < 0.001 (**) and *p* < 0.0001 (***). (*e*) Model depicting the role of Mck1 and Cdc6 in SPOC and mitotic exit regulation. Cdc6 is degraded at the G1/S phase by S-Cdk and during mitosis by the action of Mck1 with the help of M-Cdk (priming kinase for Mck1) [57,58,61]. Cdc6 starts re-accumulating at the end of mitosis, where it helps to inhibit M-Cdk activity to promote mitotic exit [58]. Cells lacking *MCK1* or bearing high levels of Cdc6 throughout mitosis are SPOC deficient. Our data show that Mck1 and Kin4 work in parallel to prevent FEAR-dependent MEN activation (see text for details).

Next, we asked if the SPOC deficiency of *mck1Δ* cells was a consequence of Cdc6 accumulation. For this, we first investigated whether increase in Cdc6 protein levels was able to engage the SPOC or promote mitotic exit. Strong overexpression of *CDC6* under the Gal1 promoter was shown to have a toxic effect due to enhanced inhibition of M-Cdk in mitosis [71,72]. However, this toxic effect was not observed upon milder overexpression [73]. To achieve moderate overexpression, we cloned *CDC6* in a multi-copy 2µ-plasmid. The presence of 2µ-*CDC6* was able to rescue the growth lethal phenotype of MEN temperature-sensitive mutants (electronic supplementary material, figure S3D) and of *lte1Δ* cells lacking *SPO12* (electronic supplementary material, figure S4A). Furthermore, 2µ*-CDC6* promoted SPOC deficiency in *kar9Δ* cells (figure 6*a,b*). The SPOC deficient phenotype was also observed upon overproduction of the essential replication defective protein Cdc6^KE^ (Cdc6-K114E) (figure 6*b*, electronic supplementary material, figure S4B) [74]. Together, these data indicate that moderate overexpression of *CDC6* phenocopies *MCK1* deletion. It also implies that Cdc6 promotes mitotic exit independently of its replication function.

From previous studies [28,73], it is known that the amino-terminal domain of Cdc6 contributes to the negative regulation of M-Cdk activity in late mitosis. To test whether the N-terminal domain and the M-Cyclin binding ^47^LxF^49^ motif of Cdc6 played a role in regulating MEN, we created a truncated version of Cdc6 lacking the N-terminal region (codons 2–49; referred as 2µ*-cdc6^NΔ^*) and a Cdc6-lxf mutant (L47A F49A; referred as 2µ*-cdc6^lxf^*), previously established to lack M-Cdk interaction and M-Cdk inhibitory activity [32,68,75]. Expression of all, wild-type *CDC6, cdc6^NΔ^* and *cdc6^lxf^* led to the survival of cells lacking endogenous *CDC6* (electronic supplementary material, figure S4B, S4D), and confirmed functionality of these constructs. The protein levels of Cdc6 expressed from the 2µ construct were similar to the levels observed for Cdc6 in *mck1Δ* cells (electronic supplementary material, figure S4C), yet Cdc6^N*Δ*^ achieved comparatively higher levels due to increased stability (electronic supplementary material figure S4C) [59].

We postulated that the SPOC deficient phenotype of *mck1Δ* cells was a consequence of increased Cdc6 levels with an intact N-terminus. To test this hypothesis, we constructed *kar9Δ mck1Δ cdc6Δ* cells expressing 2µ-*CDC6,* 2µ-*cdc6^NΔ^* or 2µ-*cdc6^lxf^.* The SPOC deficient phenotype was significantly diminished in these cells carrying 2µ-*cdc6^NΔ^* or 2µ-*cdc6^lxf^* in comparison to 2µ*-CDC6* (figure 6*c*). In addition, the overexpression of the N-terminal domain of Cdc6 (codons 1–50; 2µ*-N50-cdc6*) was still sufficient to cause SPOC deficiency in *kar9Δ* cells, yet to a lesser extent than full length 2µ-*CDC6* (figure 6*d*). These data led us to conclude that the N-terminus of Cdc6 is involved in MEN regulation. Indeed, in contrast to full-length *CDC6*, *cdc6^NΔ^* moderate overexpression did not rescue the growth phenotype of *tem1-3* cells at restrictive temperature (electronic supplementary material figure S4E). Together our data indicate that elevated levels of Cdc6 promote mitotic exit in cells with properly and misaligned spindles via its N-terminal domain.

## 3. Discussion

The SPOC is an important mitotic surveillance mechanism that responds to spindle misorientation. The kinase Kin4 is a key SPOC component that maintains the GAP complex Bfa1–Bub2 active thereby inhibiting MEN activation. Previous studies suggested that mechanisms other than Kin4 might prevent mitotic exit in cells with misoriented spindles [42,43]. In this study, we establish the role of Mck1, one of the four GSK-3 kinase homologues, as a novel component that works alongside Kin4 to engage the SPOC (figure 6*e*).

### 3.1. GSK-3 kinase Mck1 is an essential SPOC component and a mitotic exit inhibitor

Mck1, Mds1, Mrk1 and Ygk3 belong to the conserved class of GSK-3 serine/threonine kinases in *S. cerevisiae* that play important functions in the control of the meiotic and mitotic cycles [76,77]. Whereas many of these functions are uniquely played by only one of these kinases, other functions such as phosphorylation of Ime1 and Ume6 to activate transcription of early meiotic genes, are shared by two or more homologues [76,78,79]. The deletion of *MCK1* but not the other three homologues was identified as a suppressor of *KIN4* overexpression toxicity. In addition, further analysis showed that cells lacking *MCK1* but not *MDS1*, *MRK1* or *YGK3* were SPOC deficient. We reasoned that Mck1 plays a unique function in the control of SPOC that is not shared by the other three GSK-3 kinases.

Mck1 blocked MEN activation and Cdc14 release in cells with misaligned spindles, indicating that Mck1 is a SPOC component and MEN inhibitor. The analysis of *MCK1* deletion mutants, in which MEN activation was compromised, indicated that Mck1 also acts as a mitotic exit inhibitor under conditions that do not trigger spindle misorientation. This conclusion was based on the findings that lack of *MCK1* rescued the growth phenotype of MEN temperature-sensitive mutants as well as the mitotic exit defect of cells lacking the MEN activator Lte1. Growth defects in all these backgrounds were also rescued by deletion of the MEN inhibitory GAP complex Bfa1–Bub2 but not by deletion of Kin4, suggesting that Mck1 is a more potent inhibitor of mitotic exit than Kin4. Our live cell imaging and experiments using synchronized cultures showed no evidence for an inhibitory function of Mck1 on cell cycle progression in cells with properly aligned spindles at 30°C. This implies that, similar to Bfa1–Bub2 and Kin4 [41], Mck1 has no major role in delaying mitotic progression under physiological conditions but becomes essential when the spindle is misaligned. This could be explained by the presence of multiple pathways, such as FEAR, MEN and daughter enriched cell polarity proteins, that work in concert to coordinate mitotic events [1,8,46,80,81].

### 3.2. SPOC regulation by Mck1

An important consequence of SPOC activation is the prolonged anaphase duration upon spindle misalignment as a way to give cells time to correct spindle orientation before mitotic exit and cytokinesis. Cells lacking Kin4 and Bfa1–Bub2 progress through mitosis with similar timing irrespectively of spindle orientation, whereas cells lacking SWR1-complex chromatin remodellers initially engage the SPOC by a prolonged anaphase followed by mitotic slippage [44]. Based on these observations, we proposed that SPOC proteins can be sub-divided into components that ‘sense’ spindle misorientation (Kin4-pathway; comprising Kin4 and upstream regulators) and stop the MEN via Bfa1–Bub2 GAP regulation and components required to sustain the anaphase arrest (Kin4-independent regulation). The analysis of *mck1Δ* cells indicated that Mck1 had many similarities to the ‘Kin4-pathway’. For example, live cell imaging analysis showed that anaphase duration of *mck1Δ* cells with misaligned or normal aligned spindles was identical, implying that these cells cannot sense spindle misorientation. Similar to Kin4, Mck1's checkpoint function was specific to SPOC and not required for mitotic arrest imposed by the SAC that senses kinetochore–microtubule interactions in metaphase [41]. Furthermore, the SPOC deficiency of cells lacking *MCK1* was reverted after elimination of the FEAR network showing that like Kin4, a key function of Mck1 is to stop FEAR-dependent MEN activation. Both Kin4 and Mck1 inhibit MEN activation and Cdc14 release in cells with misaligned spindles. This evidence argues for a function of Mck1 in the ‘Kin4-pathway’ of SPOC. However, other results indicate that Mck1 works independently and in parallel to Kin4 to engage the SPOC. Firstly, although *MCK1* was identified in a screen for growth suppressors of *KIN4* overexpressing cells, our analysis of Kin4 protein levels, localization and function do not support a direct function of Mck1 upon Kin4. In cells lacking *MCK1*, Kin4 protein levels, localization and ability to regulate Bfa1 were unaltered indicating that Mck1 regulates SPOC by other means. Secondly, analysis of FEARless cells showed that although single deletions of *KIN4* or *MCK1* did not compromise SPOC, the double *KIN4 MCK1* deletions were SPOC deficient in this background. We favour the model that Mck1 or Kin4 maintain an inactive MEN independent of each other (figure 6*d*).

### 3.3. Cdc6 is a Mck1 substrate involved in SPOC

The SPOC function of Mck1 is kinase-dependent, indicating the involvement of one or more Mck1 substrates in mitotic control. GSK-3 family kinases often create phospho-degrons leading proteins for proteasome-dependent degradation [62]. Our data strongly suggest that Cdc6 is one of the Mck1 substrates involved in SPOC. Cdc6 is a pre-replication complex component and is targeted for degradation by Mck1 in mitosis [57,58]. In agreement with an earlier report [57], we observed higher protein levels and a strong nuclear accumulation of Cdc6 in mitotic *mck1Δ* cells irrespective of spindle orientation. Moderate overexpression of *CDC6* mimicked some phenotypes of *mck1Δ* cells*,* for example, it caused SPOC deficiency in *kar9Δ* cells and rescued the growth lethal phenotype of mitotic exit mutants. These observations support the hypothesis that higher levels of Cdc6 during mitosis promote mitotic exit in cells with misaligned spindles. Cdc6 was shown to interact with mitotic cyclin Clb2 *in vitro* and lowered the M-Cdk (Clb2-Cdc28) complex activity *in vivo* [28,32,67,68,70]. Indeed, we detected increased levels of Cdc6–Clb2 complexes in mitotic *mck1Δ* in comparison to wild-type cells, indicating that higher Cdc6 levels proportionally capture more Clb2 molecules. Cdc6 was reported to promote mitotic exit in a manner that required its N-terminal domain [28]. In agreement with this function, our data strongly support that Cdc6 drives mitotic exit in cells with misaligned spindles via its N-terminal domain and association with M-cyclin: overproduction of full length Cdc6 but not *cdc6^NΔ^* caused SPOC deficiency, and the SPOC-deficient phenotype of *mck1Δ* cells could be rescued by replacing endogenous Cdc6 with Cdc6 mutants (*cdc6^NΔ^* or *cdc6^lxf^*) unable to interact and inhibit with M-Cdk [32,68,73,75]*.* In addition, the overexpression of the N-terminal domain of Cdc6, previously shown to interact with M-Cdk [32], was sufficient to cause SPOC deficiency in *kar9Δ* cells. The SPOC-related function of Cdc6 is most likely independent of its function in replication, as overproduction of the replication inactive mutant of Cdc6 (Cdc6^KE^) caused SPOC deficiency [59,72]. As SPOC might require high M-Cdk levels for anaphase arrest [39,82], we propose that Cdc6 degradation, which initiates at G1/S phase transition and is sustained throughout mitosis, is a pre-requisite to prepare the ground for SPOC. We thus propose that Mck1 inhibits FEAR-dependent MEN activation by keeping higher M-Cdk activity through degradation of the M-Cdk inhibitor Cdc6. This hypothesis is in agreement with our data showing that higher mitotic levels of Cdc6 inactivate the SPOC in a manner that depends on its ability to downregulate Clb2–Cdk activity. MEN components including Bfa1, Cdc15 and Mob1 are targets of Clb2–Cdk and Cdc14 phosphatase [19,20,42]. Whereas Clb2–Cdk blocks MEN, FEAR-release Cdc14 was reported to promote MEN activation [6,19,20]. It is thus possible that the phosphorylation status of MEN components might change in a Mck1–Cdc6-dependent manner. Considering that only a proportion of Clb2 is captured by Cdc6 [67], the inhibition of Clb2–Cdk complex activity might be limited to changes in phosphorylation profile of a subset of components in space and time, which might be challenging to detect without more accurate or unbiased methods, such as phospho-specific antibodies or quantitative phospho-proteome analysis, respectively. It also remains to be tested if Cdc6 via its N-termin might contribute to mitotic exit through regulation of proteins other than Clb2–Cdk complexes.

The role of Mck1 in other surveillance mechanisms, like the DNA Damage Checkpoint pathway, Cell Wall Integrity pathway and heat stress response [83] has been characterized earlier. Here we have extended Mck1's function to SPOC/mitotic exit and identified Cdc6 as one Mck1 substrate that regulates SPOC. The deregulation of mitosis upon *CDC6* overexpression is not limited to *S. cerevisiae*. Its overexpression is a hallmark of certain types of tumours and it has been associated with early onset of tumorigenesis. Therefore, deciphering Mck1's substrates and, in particular, how Cdc6 is involved in the control of mitosis will greatly contribute to the understanding of the underlying molecular mechanism of mitotic regulation in depth. Interestingly, mammalian GSK-3 kinase has also been implicated in regulating spindle dynamics and spindle assembly checkpoint [84,85]. We, therefore, envisage that results obtained in yeast might provide novel insights into how spindle orientation and cell cycle progression are regulated in high eukaryotes.

## 4. Methods

### 4.1. Yeast growth conditions and synchronizations

The yeast strains and plasmids used in this study are listed in electronic supplementary material, tables S1 and S2. Standard yeast growth media and methods were used as described earlier [86]. Gene deletion and epitope tagging was done by using PCR-based methods [87,88]. Yeast cells were grown in YPDA (yeast–peptone–dextrose supplemented with 0.1 mg l^−1^ adenine) medium at 30°C unless mentioned otherwise. Temperature-sensitive strains were grown at 23°C and shifted to 37°C to arrest cells in late anaphase. Yeast strains were cultured in liquid filter sterilized synthetic complete medium for live cell imaging. To induce gene expression by the Gal1 promoter, cells were grown in medium containing 3% raffinose as the only carbon source, prior to the addition of 2% galactose. For live-cell imaging, cells were cultured in synthetic complete (SC) medium. Selection plates containing 5-fluoro-orotic acid (5-FOA, 1 mg ml^−1^) were used to select for *URA3*-based plasmid (pRS316) loss. Growth analysis to assess the effect of gene deletions was performed by drop test. For this, overnight cultures of the strains were diluted to 2 × 10^7^ cells ml^−1^ and 10-fold serial dilutions (corresponding to 10^5^; 10^4^, 10^3^, 10^2^ and 10 cells in 5 µl) were spotted on appropriate selection plates. Plates were inspected after 48–72 h. To synchronize yeast cultures in the G1 phase, cells in log phase (5 × 10^6^ cells ml^−1^) were treated with 10 µg ml^−1^ of synthetic alpha-factor (Sigma-Aldrich) for 2.5 h at 30°C or 3 h at 23°C until greater than 95% of the cells showed mating projections (shmoo formation). For metaphase arrest, cultures were incubated with 15 µg ml^−1^ of nocodazole (Sigma-Aldrich) between 2.5–4 h until greater than 90% cells were large budded with one DNA-stained region, as monitored by 4′,6-diamino-2-phenylindole (DAPI, Sigma) staining as described below.

### 4.2. Fluorescence microscopy and image analysis

For live cell imaging of cells expressing *yeGFP-TUB1*, cells were mounted on glass-bottom dishes (MatTek) coated with 6% concanavalin A-type IV (Sigma-Aldrich). Images were taken in a wide-field fluorescence imaging system (DeltaVision RT; Applied Precision) with a 100×/1.40 NA UPLS Apo UIS2 oil immersion objective lens, a charge-coupled device camera (CoolSNAP HQ/ICX285; Photometrics), a quantifiable laser module, and SoftWoRx software (Applied Precision) using an incubation chamber to maintain a constant temperature of 30°C. Live cell imaging for *CDC14-mCherry* expressing cells was performed at 30°C using a wide-field fluorescence Nikon Eclipse Ti2 Inverted Microscope equipped with a Plan Apo 100×/1.40 Oil objective and an IRIS9 Scientific CMOS camera, incubation chamber and operating Nikon NIS-Elements Imaging Software. Three z stacks of 0.5 µm thickness were taken every 90 s for 1–2 h. For analysis of still images, samples were imaged in Nikon Eclipse Ti2 Inverted Microscope. Z stacks of 0.3–0.7 µm thickness were analysed. All images were processed using the FiJi ImageJ Software [89]. Fluorescent microscopy images were analysed upon z projection of maximum signal intensity. The brightness and contrast of the images were adjusted in FiJi and no other manipulations were performed.

For budding index and DNA staining, cells were fixed in 70% ethanol and resuspended in PBS containing 1 µg ml^−1^ DAPI (Sigma-Aldrich) to visualize DNA-stained regions. The number of unbudded, small and large-budded cells was counted as well as the number and position of DAPI-stained region per cell body.

### 4.3. SPOC assays

SPOC integrity analyses were carried out using ye*GFP-TUB1 kar9Δ* strains. For population analysis of SPOC proficiency, cultures were grown at 23°C to log phase and shifted to 30°C for 6 h. Cells were fixed with 4% paraformaldehyde for 10 min at room temperature and still images were taken to observe yeGFP-Tub1. Anaphase duration of *kar9Δ yeGFP-TUB1* cells was determined by live cell imaging as the time from the beginning of spindle elongation (metaphase–anaphase transition) until spindle breakdown.

### 4.4. Protein methods and western blotting

Yeast protein extraction was carried out as previously described [87]. Briefly, cell pellets were resuspended in 1 ml of 7.5% trichloroacetic acid (TCA) in 250 mM NaOH, incubated on ice for 10 min and subsequently centrifuged at 18 407*g* for 20 min at 4°C. The resulting pellets containing precipitated proteins were resuspended in HU-DTT (200 mM Tris–HCl, pH 6.8, 8 M urea, 5% SDS, 0.1 mM EDTA, 0.005% bromo-phenol blue and 15 mg ml^−1^ DTT). Before loading on SDS-PAGE gels, samples were heated at 65°C for 15 min. To visualize phosphorylated forms of proteins, SDS-PAGE gels were run at 4°C. Western blotting was performed as described earlier [87], using nitrocellulose membrane and semi-dry transfer. Membranes were blocked with 5% low-fat milk in PBS-T (PBS containing 0.02% Tween20). Blocked membranes were incubated overnight at 4°C with primary antibody diluted in PBS-T. After washing with PBS-T, membranes were incubated for 1 h at room temperature with secondary antibody diluted in PBS-T. Proteins were visualized using enhanced chemiluminescence (ECL; Pierce; Thermo Fisher Scientific) in the ImageQuant 800 (Amersham). Detection of tubulin and Spc72 was done on stripped membranes (stripping solution: 1% SDS, 0.2 M glycine pH 2.5). Primary antibodies used were mouse anti-HA (12CA5; Sigma-Aldrich), mouse anti-Myc (Clone 9E10; Sigma-Aldrich), mouse anti-TAT1 (Sigma-Aldrich), mouse anti-GFP (clones 7.1 and 13.1; Roche), rabbit anti-Clb2 and guinea pig anti-Sic1 (Maekawa *et al*., 2007), rabbit anti-Bfa1 and rabbit anti-Spc72 (Gifts from E. Schiebel, ZMBH, Heidelberg, Germany). Secondary antibodies were goat anti-mouse, anti-rabbit or anti-guinea pig IgGs coupled to horseradish peroxidase (Jackson ImmunoResearch Laboratories, Inc.).

### 4.5. Co-immunoprecipitation experiments

Cell pellets (corresponding to 100 OD_600_) were resuspended in 300 µl of IP buffer (40 mM HEPES pH 7.5, 220 mM potassium acetate, 400 mM NaCl, 1 mM DTT) containing 100 mM β-glycerophosphate, 35 mg ml^−1^ benzamidine, 50 mM sodium fluoride, 5 mM sodium orthovanadate, 10 mM phenylmethylsulfonyl fluoride, EDTA-free protease inhibitor cocktail- PhosSTOP (Roche) and 2 µg ml^−1^ DNase I. After addition of ice-cold acid-washed glass beads (Sigma-Aldrich) to each sample, cell lysis was performed in a FastPrep FP120 Cell Disruptor (MP Biomedicals) until greater than 90% of cells were lysed as visualized by light microscope. Cell lysates were subjected to centrifugation at 4°C to eliminate cell debris and unlysed cells at maximum speed for 15 min. The resulting supernatants (total cell extract; TCE) from this were diluted equally with IP buffer containing 0.1% Tween20 and 0.2% TritonX-100 and incubated with 20 µl of GFP selector agarose beads (GFP selector, Nanotag) for 1 h at 4°C on a rotation wheel. Beads were washed five times with IP buffer with detergent. The beads were resuspended in 50 µl of 2× sample buffer (BioRad) in the presence of 5% beta-mercaptoethanol to be later analysed by western blotting.

### 4.6. Statistical analysis

To compare data sets from three independent experiments, statistical analyses were performed using the two-tailed Student's *t*-test. Data sets were considered as significantly different when *p* < 0.05 (*), *p* < 0.001 (**) and *p* < 0.0001 (***) as shown by the asterisks. Graphs were plotted in MS Excel using mean values and the error bars (depicting standard deviation (s.d.)), from three biologically independent experiments. Signal intensities for Clb2 and Cdc6-yeGFP from Co-IP immunoblots (figure 5*b*) were quantified using Fiji [89] and the levels in *mck1Δ* were normalized to those in the wild-type strain from the same experiment (figure 5*c*). The ratio of normalized intensities of Clb2 and Cdc6-yeGFP is shown (figure 5*c*).

## Data Availability

This study includes no data deposited in external repositories. Source data are available in the electronic supplementary material (electronic supplementary material, table S3) [90].

## References

[1] Caydasi AK, Pereira G. 2012 SPOC alert—when chromosomes get the wrong direction. Exp. Cell Res. **318**, 1421-1427. (10.1016/J.YEXCR.2012.03.031) 22510435

[2] Sullivan M, Morgan DO. 2007 Finishing mitosis, one step at a time. Nat. Rev. Mol. Cell Biol. **8**, 894-903. (10.1038/NRM2276) 17912263

[3] Manzano-López J, Monje-Casas F. 2020 Asymmetric cell division and replicative aging: a new perspective from the spindle poles. Curr. Genet. **66**, 719-727. (10.1007/S00294-020-01074-Y) 32266430

[4] Shou W et al. 1999 Exit from mitosis is triggered by Tem1-dependent release of the protein phosphatase Cdc14 from nucleolar RENT complex. Cell **97**, 233-244. (10.1016/S0092-8674(00)80733-3) 10219244

[5] Visintin R, Hwang ES, Amon A. 1999 Cfi1 prevents premature exit from mitosis by anchoring Cdc14 phosphatase in the nucleolus. Nature **398**, 818-823. (10.1038/19775) 10235265

[6] Stegmeier F, Visintin R, Amon A. 2002 Separase, polo kinase, the kinetochore protein Slk19, and Spo12 function in a network that controls Cdc14 localization during early anaphase. Cell **108**, 207-220. (10.1016/S0092-8674(02)00618-9) 11832211

[7] Pereira G, Schiebel E. 2003 Separase regulates INCENP-Aurora B anaphase spindle function through Cdc14. Science **302**, 2120-2124. (10.1126/SCIENCE.1091936/SUPPL_FILE/PERIERA.SOM.PDF) 14605209

[8] Rock JM, Amon A. 2009 The FEAR network. Curr. Biol. **19**, R1063-R1068. (10.1016/J.CUB.2009.10.002) 20064401 PMC3875362

[9] Visintin R, Craig K, Hwang ES, Prinz S, Tyers M, Amon A. 1998 The phosphatase Cdc14 triggers mitotic exit by reversal of Cdk-dependent phosphorylation. Mol. Cell **2**, 709-718. (10.1016/S1097-2765(00)80286-5) 9885559

[10] Jaspersen SL, Charles JF, Morgan DO. 1999 Inhibitory phosphorylation of the APC regulator Hct1 is controlled by the kinase Cdc28 and the phosphatase Cdc14. Curr. Biol. **9**, 227-236. (10.1016/S0960-9822(99)80111-0) 10074450

[11] Lee SE, Jensen S, Frenz LM, Johnson AL, Fesquet D, Johnston LH. 2001 The Bub2-dependent mitotic pathway in yeast acts every cell cycle and regulates cytokinesis. J. Cell Sci. **114**, 2345-2354. (10.1242/JCS.114.12.2345) 11493673

[12] Yoshida S, Asakawa K, Toh-e A. 2002 Mitotic exit network controls the localization of Cdc14 to the spindle pole body in *Saccharomyces cerevisiae*. Curr. Biol. **12**, 944-950. (10.1016/S0960-9822(02)00870-9) 12062061

[13] Pereira G, Manson C, Grindlay J, Schiebel E. 2002 Regulation of the Bfa1p–Bub2p complex at spindle pole bodies by the cell cycle phosphatase Cdc14p. J. Cell Biol. **157**, 367-379. (10.1083/JCB.200112085) 11970961 PMC2173300

[14] D'Amours D, Amon A. 2004 At the interface between signaling and executing anaphase—Cdc14 and the FEAR network. Genes Dev. **18**, 2581-2595. (10.1101/GAD.1247304) 15520278

[15] Shirayama M, Matsui Y, Toh-E A. 1994 The yeast TEM1 gene, which encodes a GTP-binding protein, is involved in termination of M phase. Mol. Cell Biol. **14**, 7476-7482. (10.1128/MCB.14.11.7476-7482.1994) 7935462 PMC359283

[16] Weiss EL. 2012 Mitotic exit and separation of mother and daughter cells. Genetics **192**, 1165-1202. (10.1534/GENETICS.112.145516) 23212898 PMC3512134

[17] Geymonat M, Jensen S, Johnston LH. 2002 Mitotic exit: the Cdc14 double cross. Curr. Biol. **12**, R482-R484. (10.1016/S0960-9822(02)00963-6) 12176346

[18] Jaspersen SL, Charles JF, Tinker-Kulberg RL, Morgan DO. 1998 A late mitotic regulatory network controlling cyclin destruction in *Saccharomyces cerevisiae*. Mol. Biol. Cell **9**, 2803-2817. (10.1091/MBC.9.10.2803) 9763445 PMC25555

[19] Jaspersen SL, Morgan DO. 2000 Cdc14 activates Cdc15 to promote mitotic exit in budding yeast. Curr. Biol. **10**, 615-618. (10.1016/S0960-9822(00)00491-7) 10837230

[20] König C, Maekawa H, Schiebel E. 2010 Mutual regulation of cyclin-dependent kinase and the mitotic exit network. J. Cell Biol. **188**, 351-368. (10.1083/JCB.200911128) 20123997 PMC2819678

[21] Menssen R, Neutzner A, Seufert W. 2001 Asymmetric spindle pole localization of yeast Cdc15 kinase links mitotic exit and cytokinesis. Curr. Biol. **11**, 345-350. (10.1016/S0960-9822(01)00095-1) 11267871

[22] Visintin R, Amon A. 2001 Regulation of the mitotic exit protein kinases Cdc15 and Dbf2. Mol. Biol. Cell **12**, 2961. (10.1091/MBC.12.10.2961) 11598184 PMC60148

[23] Rock JM et al. 2013 Activation of the yeast Hippo pathway by phosphorylation-dependent assembly of signaling complexes. Science **340**, 871-875. (10.1126/SCIENCE.1235822) 23579499 PMC3884217

[24] Lee SE, Frenz LM, Wells NJ, Johnson AL, Johnston LH. 2001 Order of function of the budding-yeast mitotic exit-network proteins Tem1, Cdc15, Mob1, Dbf2, and Cdc5. Curr. Biol. **11**, 784-788. (10.1016/S0960-9822(01)00228-7) 11378390

[25] Campbell IW, Zhou X, Amon A. 2019 The mitotic exit network integrates temporal and spatial signals by distributing regulation across multiple components. Elife **8**, e41139. (10.7554/ELIFE.41139) 30672733 PMC6363386

[26] Meitinger F, Palani S, Pereira G. 2012 The power of MEN in cytokinesis. Cell Cycle **11**, 219-228. (10.4161/CC.11.2.18857) 22189712

[27] Manzano-López J, Monje-Casas F. 2020 The multiple roles of the Cdc14 phosphatase in cell cycle control. Int. J. Mol. Sci. **21**, 709. (10.3390/IJMS21030709) 31973188 PMC7038166

[28] Calzada A, Sacristán M, Sánchez E, Bueno A. 2001 Cdc6 cooperates with Sic1 and Hct1 to inactivate mitotic cyclin-dependent kinases. Nature **412**, 355-358. (10.1038/35085610) 11460169

[29] Schwab M, Neutzner M, Möcker D, Seufert W. 2001 Yeast Hct1 recognizes the mitotic cyclin Clb2 and other substrates of the ubiquitin ligase APC. EMBO J. **20**, 5165. (10.1093/EMBOJ/20.18.5165) 11566880 PMC125620

[30] Wäsch R, Cross FR. 2002 APC-dependent proteolysis of the mitotic cyclin Clb2 is essential for mitotic exit. Nature **418**, 556-562. (10.1038/nature00856) 12152084

[31] Bell SP, Dutta A. 2003 DNA replication in eukaryotic cells. Ann. Rev. Biochem. **71**, 333-374. (10.1146/annurev.biochem.71.110601.135425) 12045100

[32] Elsasser S, Lou F, Wang B, Campbell JL, Jong A. 1996 Interaction between yeast Cdc6 protein and B-type cyclin/Cdc28 kinases. Mol. Biol. Cell **7**, 1723-1735. (10.1091/MBC.7.11.1723) 8930895 PMC276021

[33] D'Aquino KE, Monje-Casas F, Paulson J, Reiser V, Charles GM, Lai L, Shokat KM, Amon A. 2005 The protein kinase Kin4 inhibits exit from mitosis in response to spindle position defects. Mol. Cell **19**, 223-234. (10.1016/J.MOLCEL.2005.06.005) 16039591

[34] Pereira G, Schiebel E. 2005 Kin4 kinase delays mitotic exit in response to spindle alignment defects. Mol. Cell **19**, 209-221. (10.1016/J.MOLCEL.2005.05.030) 16039590

[35] Hu F, Wang Y, Liu D, Li Y, Qin J, Elledge SJ. 2001 Regulation of the Bub2/Bfa1 GAP complex by Cdc5 and cell cycle checkpoints. Cell **107**, 655-665. (10.1016/S0092-8674(01)00580-3) 11733064

[36] Caydasi AK, Micoogullari Y, Kurtulmus B, Palani S, Pereira G. 2014 The 14-3-3 protein Bmh1 functions in the spindle position checkpoint by breaking Bfa1 asymmetry at yeast centrosomes. Mol. Biol. Cell **25**, 2143-2151. (10.1091/MBC.E14-04-0890) 24850890 PMC4091827

[37] Caydasi AK, Pereira G. 2009 Spindle alignment regulates the dynamic association of checkpoint proteins with yeast spindle pole bodies. Dev. Cell **16**, 146-156. (10.1016/J.DEVCEL.2008.10.013) 19154725

[38] Monje-Casas F, Amon A. 2009 Cell polarity determinants establish asymmetry in MEN signaling. Dev. Cell **16**, 132-145. (10.1016/J.DEVCEL.2008.11.002) 19154724 PMC2713012

[39] Caydasi AK, Lohel M, Grünert G, Dittrich P, Pereira G, Ibrahim B. 2012 A dynamical model of the spindle position checkpoint. Mol. Syst. Biol. **8**, 582. (10.1038/MSB.2012.15) 22580890 PMC3377990

[40] Pereira G, Höfken T, Grindlay J, Manson C, Schiebel E. 2000 The Bub2p spindle checkpoint links nuclear migration with mitotic exit. Mol. Cell **6**, 1-10. (10.1016/S1097-2765(05)00017-1) 10949022

[41] Caydasi AK, Kurtulmus B, Orrico MIL, Hofmann A, Ibrahim B, Pereira G. 2010 Elm1 kinase activates the spindle position checkpoint kinase Kin4. J. Cell Biol. **190**, 975. (10.1083/JCB.201006151) 20855503 PMC3101594

[42] Caydasi AK, Khmelinskii A, Duenas-Sanchez R, Kurtulmus B, Knop M, Pereira G. 2017 Temporal and compartment-specific signals coordinate mitotic exit with spindle position. Nat. Commun. **8**, 1-14. (10.1038/ncomms14129) 28117323 PMC5286211

[43] Falk JE, Campbell IW, Joyce K, Whalen J, Seshan A, Amon A. 2016 LTE1 promotes exit from mitosis by multiple mechanisms. Mol. Biol. Cell **27**, 3991-4001. (10.1091/MBC.E16-08-0563/ASSET/IMAGES/LARGE/MBC-27-3991-G008.JPEG) 27798238 PMC5156540

[44] Caydasi AK, Khmelinskii A, Darieva Z, Kurtulmus B, Knop M, Pereira G. 2020 SWR1 chromatin remodeling complex prevents mitotic slippage during spindle position checkpoint arrest. *bioRxiv* 749440. (10.1101/749440) PMC9930528 36542480

[45] Kocakaplan D, Karabürk H, Dilege C, Kirdok I, Bektaş ŞN, Caydasi AK. 2021 Protein phosphatase 1 in association with bud14 inhibits mitotic exit in *Saccharomyces cerevisiae*. Elife **10**, e72833. (10.7554/ELIFE.72833) 34633288 PMC8577847

[46] Scarfone I, Piatti S. 2015 Coupling spindle position with mitotic exit in budding yeast: the multifaceted role of the small GTPase Tem1. Small GTPases **6**, 196-201. (10.1080/21541248.2015.1109023) 26507466 PMC4905282

[47] Miller RK, Rose MD. 1998 Kar9p is a novel cortical protein required for cytoplasmic microtubule orientation in yeast. J. Cell Biol. **140**, 377. (10.1083/JCB.140.2.377) 9442113 PMC2132572

[48] Daum JR, Gomez-Ospina N, Winey M, Burke DJ. 2000 The spindle checkpoint of *Saccharomyces cerevisiae* responds to separable microtubule-dependent events. Curr. Biol. **10**, 1375-1378. (10.1016/S0960-9822(00)00780-6) 11084338

[49] Drechsler H, Tan AN, Liakopoulos D. 2015 Yeast GSK-3 kinase regulates astral microtubule function through phosphorylation of the microtubule-stabilizing kinesin Kip2. J. Cell Sci. **128**, 3910-3921. (10.1242/JCS.166686/-/DC1) 26395399 PMC4657329

[50] McQueen J, van Dyk D, Young B, Loewen C, Measday V. 2012 The Mck1 GSK-3 kinase inhibits the activity of Clb2–Cdk1 post-nuclear division. Cell Cycle **11**, 3421. (10.4161/CC.21731) 22918234 PMC3466553

[51] Maekawa H, Priest C, Lechner J, Pereira G, Schiebel E. 2007 The yeast centrosome translates the positional information of the anaphase spindle into a cell cycle signal. J. Cell Biol. **179**, 423-436. (10.1083/JCB.200705197) 17967947 PMC2064790

[52] Bertazzi DT, Kurtulmus B, Pereira G. 2011 The cortical protein Lte1 promotes mitotic exit by inhibiting the spindle position checkpoint kinase Kin4. J. Cell Biol. **193**, 1033-1048. (10.1083/JCB.201101056) 21670215 PMC3115795

[53] Molk JN, Schuyler SC, Liu JY, Evans JG, Salmon ED, Pellman D, Bloom K. 2004 The differential roles of budding yeast Tem1p, Cdc15p, and Bub2p protein dynamics in mitotic exit. Mol. Biol. Cell **15**, 1519. (10.1091/MBC.E03-09-0708) 14718561 PMC379252

[54] Visintin R, Stegmeier F, Amon A. 2003 The role of the polo kinase Cdc5 in controlling Cdc14 localization. Mol. Biol. Cell **14**, 4486. (10.1091/MBC.E03-02-0095) 14551257 PMC266767

[55] Falk JE, Chan LY, Amon A. 2011 Lte1 promotes mitotic exit by controlling the localization of the spindle position checkpoint kinase Kin4. Proc. Natl Acad. Sci. USA **108**, 12 584-12 590. (10.1073/PNAS.1107784108) 21709215 PMC3150932

[56] Shirayama M, Matsui Y, Tanaka K, Toh-E A. 1994 Isolation of a CDC25 family gene, MSI2/LTE1, as a multicopy suppressor of ira1. Yeast **10**, 451-461. (10.1002/YEA.320100404) 7941731

[57] Ikui AE, Rossio V, Schroeder L, Yoshida S. 2012 A yeast GSK-3 kinase Mck1 promotes Cdc6 degradation to inhibit DNA re-replication. PLoS Genet. **8**, e1003099. (10.1371/JOURNAL.PGEN.1003099) 23236290 PMC3516531

[58] Al-Zain A, Schroeder L, Sheglov A, Ikui AE. 2015 Cdc6 degradation requires phosphodegron created by GSK-3 and Cdk1 for SCFCdc4 recognition in *Saccharomyces cerevisiae*. Mol. Biol. Cell **26**, 2609-2619. (10.1091/MBC.E14-07-1213/ASSET/IMAGES/LARGE/MBC-26-2609-G005.JPEG) 25995377 PMC4501359

[59] Drury LS, Perkins G, Diffley JFX. 1997 The Cdc4/34/53 pathway targets Cdc6p for proteolysis in budding yeast. EMBO J **16**, 5966-5976. (10.1093/EMBOJ/16.19.5966) 9312054 PMC1170227

[60] Drury LS, Perkins G, Diffley JFX. 2000 The cyclin-dependent kinase Cdc28p regulates distinct modes of Cdc6p proteolysis during the budding yeast cell cycle. Curr. Biol. **10**, 231-240. (10.1016/S0960-9822(00)00355-9) 10712901

[61] Perkins G, Drury LS, Diffley JFX. 2001 Separate SCFCDC4 recognition elements target Cdc6 for proteolysis in S phase and mitosis. EMBO J **20**, 4836. (10.1093/EMBOJ/20.17.4836) 11532947 PMC125267

[62] Fiol CJ, Mahrenholz AM, Wang Y, Roeske RW, Roach PJ. 1987 Formation of protein kinase recognition sites by covalent modification of the substrate. Molecular mechanism for the synergistic action of casein kinase II and glycogen synthase kinase 3. J. Biol. Chem. **262**, 14 042-14 048. (10.1016/S0021-9258(18)47901-X) 2820993

[63] Luo KQ, Elsasser S, Chang DC, Campbell JL. 2003 Regulation of the localization and stability of Cdc6 in living yeast cells. Biochem. Biophys. Res. Commun. **306**, 851-859. (10.1016/S0006-291X(03)01082-9) 12821120

[64] Chen Y, Hennessy KM, Botstein D, Tye BK. 1992 CDC46/MCM5, a yeast protein whose subcellular localization is cell cycle-regulated, is involved in DNA replication at autonomously replicating sequences. Proc. Natl Acad. Sci. USA **89**, 10459. (10.1073/PNAS.89.21.10459) 1438234 PMC50358

[65] Dalton S, Whitbread L. 1995 Cell cycle-regulated nuclear import and export of Cdc47, a protein essential for initiation of DNA replication in budding yeast. Proc. Natl Acad. Sci. USA **92**, 2514-2518. (10.1073/PNAS.92.7.2514) 7708676 PMC42248

[66] McInerny CJ, Partridge JF, Mikesell GE, Creemer DP, Breeden LL. 1997 A novel Mcm1-dependent element in the SWI4, CLN3, CDC6, and CDC47 promoters activates M/G1-specific transcription. Genes Dev. **11**, 1277-1288. (10.1101/GAD.11.10.1277) 9171372

[67] Mimura S, Seki T, Tanaka S, Diffley JFX. 2004 Phosphorylation-dependent binding of mitotic cyclins to Cdc6 contributes to DNA replication control. Nature **431**, 1118-1123. (10.1038/NATURE03024) 15496876

[68] Örd M, Möll K, Agerova A, Kivi R, Faustova I, Venta R, Valk E, Loog M. 2019 Multisite phosphorylation code of CDK. Nat. Struct. Mol. Biol. **26**, 649-658. (10.1038/S41594-019-0256-4) 31270471 PMC6614033

[69] Philip J, Örd M, Silva A, Singh S, Diffley JF, Remus D, Loog M, Ikui AE. 2022 Cdc6 is sequentially regulated by PP2A-Cdc55, Cdc14, and Sic1 for origin licensing in *S. cerevisiae*. Elife **11**, e74437. (10.7554/ELIFE.74437) 35142288 PMC8830886

[70] Sánchez M, Calzada A, Bueno A. 1999 The Cdc6 protein is ubiquitinated *in vivo* for proteolysis in *Saccharomyces cerevisiae*. J. Biol. Chem. **274**, 9092-9097. (10.1074/JBC.274.13.9092) 10085159

[71] Boronat S, Campbell JL. 2007 Mitotic Cdc6 stabilizes anaphase-promoting complex substrates by a partially Cdc28-independent mechanism, and this stabilization is suppressed by deletion of Cdc55. Mol. Cell Biol. **27**, 1158-1171. (10.1128/MCB.01745-05) 17130241 PMC1800676

[72] Bueno A, Russell P. 1992 Dual functions of CDC6: a yeast protein required for DNA replication also inhibits nuclear division. EMBO J. **11**, 2167-2176. (10.1002/J.1460-2075.1992.TB05276.X) 1600944 PMC556684

[73] Archambault V, Li CX, Tackett AJ, Wäsch R, Chait BT, Rout MP, Cross FR. 2003 Genetic and biochemical evaluation of the importance of Cdc6 in regulating mitotic exit. Mol. Biol. Cell **14**, 4592-4604. (10.1091/MBC.E03-06-0384) 12960422 PMC313736

[74] Weinreich M, Liang C, Stillman B. 1999 The Cdc6p nucleotide-binding motif is required for loading Mcm proteins onto chromatin. Proc. Natl Acad. Sci. USA **96**, 441. (10.1073/PNAS.96.2.441) 9892652 PMC15155

[75] Örd M, Venta R, Möll K, Valk E, Loog M. 2019 Cyclin-specific docking mechanisms reveal the complexity of M-CDK function in the cell cycle. Mol. Cell **75**, 76. (10.1016/J.MOLCEL.2019.04.026) 31101497 PMC6620034

[76] Neigeborn L, Mitchell AP. 1991 The yeast MCK1 gene encodes a protein kinase homolog that activates early meiotic gene expression. Genes Dev. **5**, 533-548. (10.1101/GAD.5.4.533) 2010083

[77] Andoh T, Hirata Y, Kikuchi A. 2000 Yeast glycogen synthase kinase 3 is involved in protein degradation in cooperation with Bul1, Bul2, and Rsp5. Mol. Cell Biol. **20**, 6712. (10.1128/MCB.20.18.6712-6720.2000) 10958669 PMC86186

[78] Rubin-Bejerano I, Sagee S, Friedman O, Pnueli L, Kassir Y. 2004 The *in vivo* activity of Ime1, the key transcriptional activator of meiosis-specific genes in *Saccharomyces cerevisiae*, is inhibited by the cyclic AMP/protein kinase A signal pathway through the glycogen synthase kinase 3-beta homolog Rim11. Mol. Cell Biol. **24**, 6967-6979. (10.1128/MCB.24.16.6967-6979.2004) 15282298 PMC479714

[79] Vershon AK, Pierce M. 2000 Transcriptional regulation of meiosis in yeast. Curr. Opin. Cell Biol. **12**, 334-339. (10.1016/S0955-0674(00)00104-6) 10801467

[80] Nelson SA, Cooper JA. 2007 A novel pathway that coordinates mitotic exit with spindle position. Mol. Biol. Cell **18**, 3440-3450. (10.1091/MBC.E07-03-0242) 17615297 PMC1951770

[81] Höfken T, Schiebel E. 2002 A role for cell polarity proteins in mitotic exit. EMBO J **21**, 4851-4862. (10.1093/EMBOJ/CDF481) 12234925 PMC126280

[82] Howell RSM, Klemm C, Thorpe PH, Csikasz-Nagy A. 2020 Unifying the mechanism of mitotic exit control in a spatiotemporal logical model. PLoS Biol. **18**, e3000917. (10.1371/JOURNAL.PBIO.3000917) 33180788 PMC7685450

[83] Delgado NS, Toczyski DP. 2019 Mck1 kinase is a new player in the DNA damage checkpoint pathway. PLoS Genet. **15**, e1008372. (10.1371/JOURNAL.PGEN.1008372) 31671089 PMC6822710

[84] Wakefield JG, Stephens DJ, Tavaré JM. 2003 A role for glycogen synthase kinase-3 in mitotic spindle dynamics and chromosome alignment. J. Cell Sci. **116**, 637-646. (10.1242/JCS.00273) 12538764

[85] Rashid MS, Mazur T, Ji W, Liu ST, Taylor WR. 2018 Analysis of the role of GSK3 in the mitotic checkpoint. Sci. Rep. **8**, 1-16. (10.1038/s41598-018-32435-w) 30250048 PMC6155330

[86] Sherman F. 1991 [1] Getting started with yeast. Methods Enzymol. **194**, 3-21. (10.1016/0076-6879(91)94004-V) 2005794

[87] Janke C et al. 2004 A versatile toolbox for PCR-based tagging of yeast genes: new fluorescent proteins, more markers and promoter substitution cassettes. Yeast **21**, 947-962. (10.1002/YEA.1142) 15334558

[88] Knop M, Siegers K, Pereira G, Zachariae W, Winsor B, Nasmyth K, Schiebel E. 1999 Epitope tagging of yeast genes using a PCR-based strategy: more tags and improved practical routines. Yeast **15**, 963-972. (10.1002/(sici)1097-0061(199907)15:10b<963::aid-yea399>3.0.co;2-w) 10407276

[89] Schindelin J et al. 2012 Fiji: an open-source platform for biological-image analysis. Nat. Methods **9**, 676-682. (10.1038/NMETH.2019) 22743772 PMC3855844

[90] Rathi S, Polat I, Pereira G. 2022 Data from: The budding yeast GSK-3 homologue Mck1 is an essential component of the spindle position checkpoint. Figshare. (10.6084/m9.figshare.c.6261880) PMC9627454 36321416

